# Association of Maternal Caffeine Consumption During Pregnancy With Child Growth

**DOI:** 10.1001/jamanetworkopen.2022.39609

**Published:** 2022-10-31

**Authors:** Jessica L. Gleason, Rajeshwari Sundaram, Susanna D. Mitro, Stefanie N. Hinkle, Stephen E. Gilman, Cuilin Zhang, Roger B. Newman, Kelly J. Hunt, Daniel W. Skupski, William A. Grobman, Michael Nageotte, Morgan Robinson, Kurunthachalam Kannan, Katherine L. Grantz

**Affiliations:** 1 Epidemiology Branch, Division of Population Health Research, Division of Intramural Research, Eunice Kennedy Shriver National Institute of Child Health and Human Development, Bethesda, Maryland; 2 Biostatistics and Bioinformatics Branch, Division of Population Health Research, Division of Intramural Research, Eunice Kennedy Shriver National Institute of Child Health and Human Development, Bethesda, Maryland; 3 Department of Biostatistics, Epidemiology and Informatics, Perelman School of Medicine, University of Pennsylvania, Philadelphia; 4 Social and Behavioral Sciences Branch, Division of Population Health Research, Division of Intramural Research, Eunice Kennedy Shriver National Institute of Child Health and Human Development, Bethesda, Maryland; 5 Department of Mental Health, Johns Hopkins Bloomberg School of Public Health, Baltimore, Maryland; 6 Bia-Echo Asia Centre for Reproductive Longevity & Equality, Department of Obstetrics and Gynecology, Yong Loo Lin School of Medicine, National University of Singapore, Singapore; 7 Department of Obstetrics and Gynecology, Medical University of South Carolina, Charleston; 8 Department of Public Health Sciences, Medical University of South Carolina, Charleston; 9 Weill Cornell Medicine and New York Presbyterian Queens, New York, New York; 10 Department of Obstetrics and Gynecology, The Ohio State University, Columbus; 11 Miller Children’s and Women’s Hospital, Long Beach, California; 12 Department of Pediatrics and Department of Environmental Medicine, New York University School of Medicine, New York

## Abstract

**Importance:**

Greater caffeine consumption in pregnancy is associated with reduced birth size, but potential associations with childhood growth are unclear.

**Objective:**

To evaluate the associations of pregnancy caffeine and paraxanthine measures with child growth in a contemporary cohort with low caffeine consumption and a historical cohort with high caffeine consumption.

**Design, Setting, and Participants:**

The Environmental Influences on Child Health Outcomes cohort of the National Institute of Child Health and Human Development Fetal Growth Studies (ECHO-FGS; 10 sites, 2009-2013) was a pregnancy cohort with 1 child measurement between ages 4 and 8 years (follow-up in 2017-2019). The Collaborative Perinatal Project (CPP) was a pregnancy cohort (12 sites, 1959-1965) with child follow-up through 8 years (1960-1974). The current secondary analysis was conducted in 2021 and 2022.

**Exposures:**

Concentrations of caffeine and its primary metabolite, paraxanthine, were quantified from plasma (ECHO-FGS) and serum (CPP) collected in the first trimester. Cut points for analyses were defined by quartiles in ECHO-FGS and quintiles in CPP.

**Main Outcomes and Measures:**

Child *z* scores for body mass index, weight, and height were evaluated, as well as fat mass index and percentage and obesity risk measured at 1 time between age 4 and 8 years in ECHO-FGS. In a secondary analysis of the CPP cohort, child *z* scores and obesity risk longitudinally through age 8 years were evaluated.

**Results:**

In ECHO-FGS (median caffeine intake <50 mg/d), 788 children (mean [SD] age, 6.8 [1.0] years; 411 boys [52.2%]) of women in the fourth vs first quartile of plasma caffeine concentrations had lower height *z* scores (β = −0.21; 95% CI, −0.41 to −0.02), but differences in weight *z* scores were only observed in the third quartile (β = −0.27; 95% CI, −0.47 to −0.07). In CPP, beginning at age 4 years, 1622 children (805 boys [49.7%]) of women in the highest caffeine quintile group had lower height *z* scores than their peers from the lowest group, with the gap widening with each successive year of age (β = −0.16 [95% CI, −0.31 to −0.01] at 4 years; β = −0.37 [95% CI, −0.57 to −0.16] at 8 years). There were slight reductions in weight at ages 5 to 8 years for children in the third vs first caffeine quintile (β = −0.16 to −0.22). Results were consistent for paraxanthine concentrations in both cohorts.

**Conclusions and Relevance:**

Intrauterine exposure to increasing levels of caffeine and paraxanthine, even in low amounts, was associated with shorter stature in early childhood. The clinical implication of reductions in height and weight is unclear; however, the reductions were apparent even with levels of caffeine consumption below clinically recommended guidelines of less than 200 mg per day.

## Key Points

**Question:**

Is maternal caffeine consumption associated with child growth, and are such associations present in low-consumption groups?

**Findings:**

In this cohort study, at ages 4 to 8 years, children of women with low measured caffeine and paraxanthine during pregnancy were shorter than the children of women who consumed no caffeine during pregnancy, with increasing gaps in height in a historical cohort through age 8 years. There were no clear patterns of weight or body mass index changes.

**Meaning:**

Although the clinical implications are unclear for relatively small observed differences, these findings suggest that small amounts of daily maternal caffeine consumption are associated with shorter stature in their offspring that persist into childhood.

## Teaser

This cohort study evaluates the association of caffeine consumption during pregnancy and paraxanthine measures with child growth.

## Introduction

Maternal caffeine consumption during pregnancy, even in modest amounts (eg, 50 mg or one-half cup of coffee per day), is associated with lower birth weight and higher rates of birth weight below 2500 g.^1,2^ These decreases in birth weight were attributed to shorter birth length and less lean tissue mass in the National Institute of Child Health and Human Development (NICHD) Fetal Growth Studies.^3^ The potential mechanism is unclear, but caffeine is a neural stimulant not metabolized by the fetus that accumulates in fetal tissue.^4^ Given that approximately 8 in 10 US pregnant women consume caffeine,^5^ it is important to determine whether in utero caffeine exposure has long-term growth implications in offspring.

Studies^6,7,8,9,10^ using self-reported caffeine consumption during pregnancy have suggested associations with excess weight gain in infancy, higher body mass index (BMI), and risk for obesity in children. Most of these studies^6,7,8^ were conducted in regions with high mean caffeine intake among participants. Therefore, it is unknown whether lower doses of caffeine are biologically relevant to child growth. The aim of this cohort study was to follow up our previous findings^3^ in a low-consumption cohort to investigate whether the association of maternal caffeine consumption with smaller neonatal anthropometry of offspring at birth persisted into childhood by investigating differences in child weight, BMI, and fat mass across different maternal blood concentrations of caffeine and its primary metabolite, paraxanthine. A subset of offspring from the NICHD Fetal Growth Studies–Singletons were subsequently followed in the Environmental Influences on Child Health Outcomes (ECHO-FGS) study. We also performed a secondary analysis in a historical cohort with higher caffeine exposure (estimated median 2 cups of coffee per day).

## Methods

### Sample and Anthropometric Measures

The ECHO-FGS study (10 sites, 2009-2013; follow-up in 2017-2019) included 1116 mother-child pairs from the Eunice Kennedy Shriver NICHD Fetal Growth Studies–Singletons cohort.^11^ Women in this cohort included women without obesity at lower risk for fetal growth abnormalities and a group with obesity.^12^ Children aged 4 to 8 years completed an in-person ECHO-FGS examination with measures of height, weight, and maternal plasma caffeine and paraxanthine concentrations measured during pregnancy. Fat mass was assessed using a bioelectric impedance analyzer (Quantum; RJL Systems, Inc). Percentage body fat was computed as [fat mass / (fat + fat-free mass)] × 100, and fat mass index was computed as fat mass in kilograms divided by height in meters squared.^13,14^

The Collaborative Perinatal Project (CPP) was a prospective cohort of pregnant women (12 sites, 1959-1965; follow-up in 1960-1974) with child follow-up measures of height and weight at birth; 4, 8, and 12 months; and 3, 4, 7, and 8 years. To maximize comparability to ECHO-FGS, this analysis included singleton children of nonsmoking women with height or weight data for at least 1 official follow-up visit. For additional details on the CPP and procedures for child measurement, see the eMethods in the Supplement. The original procedures of the ECHO-FGS were reviewed by institutional review boards at NICHD, data coordinating centers, and individual sites. Study participants provided written consent prior to data collection. The CPP data are deidentified for public use and considered exempt from ethics review in accordance with 45 CFR §46. This study followed the Strengthening the Reporting of Observational Studies in Epidemiology (STROBE) reporting guideline cohort studies.

To compare growth measures across ages, we calculated age-normalized and sex-normalized BMI and *z* scores for BMI, height, and weight, using the World Health Organization growth charts for ages 0 to 23 months and Centers for Disease Control and Prevention growth charts for children older than 24 months.^15,16^ For the sake of comparison, we translated *z* scores to approximate differences in standardized weight or height, using the Centers for Disease Control and Prevention reference charts at age 7 years, because this was the average age of measurement for ECHO-FGS and coincided with an official study visit in CPP. We explored overweight and obesity using age-specific and sex-specific cut points from the International Obesity Task Force for all children aged 2 years or older.^17^

### Caffeine and Paraxanthine

Caffeine has a short half-life and is primarily metabolized to paraxanthine in as little as 3 hours in pregnant women in their first trimester, with slowing metabolism up to 10 hours by the third trimester.^18^ Thus, we evaluated both caffeine and paraxanthine in association with child growth. In ECHO-FGS, caffeine and paraxanthine were measured in first-trimester plasma samples (mean [SD], 12.6 [1.0] gestational weeks). Extraction was accomplished via hybrid solid phase extraction, and quantification of caffeine was performed on an ABSCIEX 5500 (Applied Biosystems). The limits of detection of caffeine and paraxanthine through the analytical method were 0.55 and 0.72 ng/mL, respectively, and limits of quantitation were 1.85 and 2.39 ng/mL, respectively. In CPP, caffeine and paraxanthine were measured in stored serum samples obtained before 20 weeks’ gestation (mean [SD], 11.2 [2.9] weeks) and quantified using high-performance liquid chromatography in 1998 as part of a case-control study.^19^ The limits of detection and quantitation were 25 and 50 ng/mL, respectively. Of note, plasma and serum caffeine have identical reference ranges in laboratory tests and there is no evidence to suggest values would differ between components.^20^

We categorized caffeine and paraxanthine concentrations into quartiles in ECHO-FGS on the basis of their distributions in the cohort, consistent with our previous work on caffeine and neonatal anthropometry.^3^ For CPP, we categorized caffeine and paraxanthine into quintiles to reflect the wider distribution of measures in this cohort. For all analyses, the first quartile or quintile was considered the reference group because levels were so low.

### Covariates

We adjusted for the same constructs in both cohorts: maternal age, parity, socioeconomic status, self-reported race and ethnicity as a proxy for unmeasured social factors, married or living with partner, smoking status, and study site. Most covariates were similar between cohorts, but in CPP, we restricted to nonsmokers, and in ECHO-FGS, where women were mostly nonsmokers, we adjusted for plasma cotinine level at 10 to 13 weeks of gestation to account for primary and secondary exposure to cigarette smoke. Additionally, in the CPP, socioeconomic status was determined using the socioeconomic index, which combined parental education, employment, and household income and is interpreted as a percentile score of socioeconomic status.^21^ In ECHO-FGS, we adjusted for maternal education. Given the heritability of height and weight patterns, we adjusted child height analyses for maternal height and child weight analyses for maternal prepregnancy weight, prioritizing measured maternal values over self-report. All other analyses were adjusted for prepregnancy BMI collected at enrollment.

### Statistical Analysis

Characteristics were compared between participants across quartiles and quintiles of caffeine concentrations for each cohort using 2-sided *t* tests and χ^2^ tests for continuous and categorical variables, respectively. To evaluate the association between caffeine and paraxanthine quartiles or quintiles and child growth, we fit generalized linear mixed models for each growth parameter, including *z* scores for height, weight, BMI; fat mass index; and fat mass percentile. The results of these models are β values, which indicate the mean difference in an outcome from a specified reference value (ie, the first quartile or quintile). We adjusted for child age in our models, including interaction terms between caffeine or paraxanthine and age, to allow for flexibility in the association between caffeine biomarkers and growth over time. To test for possible effect modification by child sex, we added 2-way interaction terms. We included additional components to models in CPP to account for repeated measurements over time (eMethods in the Supplement). We used Poisson regression with a robust error variance to investigate associations of caffeine and paraxanthine with the risk of overweight and obesity at any point between ages 2 to 8 years, based on International Obesity Task Force cut points.^17^ Statistical significance was set at *P* < .05 for all analyses. The current analysis was conducted in 2021 and 2022. Data were analyzed using SAS statistical software version 9.4 (SAS Institute).

## Results

Participant characteristics for ECHO-FGS (788 children; 411 boys [52.2%]) are presented in Table 1. The cohort was racially and ethnically diverse by design, including 236 non-Hispanic White women (30.0%), 247 non-Hispanic Black women (31.4%), 201 Hispanic women (25.5%), and 104 Asian or Pacific Islander women (13.2%). Women in the lowest caffeine concentration category tended to be younger, nulliparous, and identified as non-Hispanic Black, with a lower percentage being married or partnered or in higher education or socioeconomic categories. The mean (SD) age of children was 6.8 (1.0) years, and 23.7% (187 children) had overweight or obesity. Participant characteristics for the 1622 children in the CPP cohort (805 boys [49.7%]) are presented in eTable 1 in the Supplement.

**Table 1.** Maternal and Child Characteristics, Environmental Influences on Child Outcomes Cohort of the National Institute of Child Health and Human Development Fetal Growth Studies–Singletons

Characteristics^a^	Overall (N = 788)	Maternal caffeine concentration quartile, ng/mL			
		<25.4 (n = 197)	25.4-146.6 (n = 197)	146.7-575.2 (n = 197)	≥575.3 (n = 197)
Maternal age, mean (SD), y	28.4 (5.7)	27.1 (5.7)	28.8 (5.8)	27.9 (5.5)	30.1 (5.4)
Maternal education					
Less than high school	90 (11.4)	24 (11.0)	23 (11.9)	25 (12.7)	18 (10.1)
High school diploma or general equivalency diploma	129 (16.4)	52 (23.7)	26 (13.4)	33 (16.8)	18 (10.1)
Some college or associate’s degree	234 (29.7)	66 (30.1)	53 (27.3)	56 (28.4)	59 (33.2)
Bachelor’s degree	193 (24.5)	44 (20.1)	54 (27.8)	49 (24.9)	46 (25.8)
Master’s or advanced degree	142 (18.0)	33 (15.1)	38 (19.6)	34 (17.3)	37 (20.8)
Parity (predelivery)					
0	356 (45.2)	122 (55.7)	82 (42.3)	80 (40.6)	72 (40.5)
1	269 (34.1)	57 (26.0)	76 (39.2)	79 (40.1)	57 (32.0)
≥2	163 (20.7)	40 (18.3)	36 (18.6)	38 (19.3)	49 (27.5)
Race and ethnicity					
Asian and Pacific Islander	104 (13.2)	36 (16.4)	25 (12.9)	17 (8.6)	26 (14.6)
Black	247 (31.4)	109 (49.8)	57 (29.4)	52 (26.4)	29 (16.3)
Hispanic	201 (25.5)	42 (19.2)	57 (29.4)	62 (31.5)	40 (22.5)
White	236 (30.0)	32 (14.6)	55 (28.4)	66 (33.5)	83 (46.6)
Married	566 (71.8)	131 (59.8)	142 (73.2)	148 (75.1)	145 (81.5)
Maternal weight, mean (SD), kg	67.9 (15.1)	68.9 (16.7)	67.2 (15.0)	68.4 (14.3)	66.6 (13.8)
Maternal height, mean (SD), m	162.8 (7.1)	1.63 (0.07)	1.63 (0.07)	1.63 (0.07)	1.62 (0.07)
Maternal prepregnancy body mass index, mean (SD)^b^	25.6 (5.3)	25.7 (5.6)	25.3 (5.2)	25.9 (5.2)	25.3 (5.0)
Child sex, male	411 (52.2)	115 (52.5)	109 (56.2)	95 (48.2)	92 (51.7)

^a^ Includes liveborn singletons with height or weight measurements and plasma caffeine or paraxanthine measured in pregnancy.

^b^ Body mass index is calculated as weight in kilograms divided by height in meters squared.

### ECHO-FGS Cohort

The median (IQR) maternal caffeine and paraxanthine concentrations were 168.5 (29.5 to 650.5) ng/mL and 73.8 (15.3 to 236.3) ng/mL, respectively, which roughly translates to median consumption of less than 50 mg per day,^22^ consistent with their reported mean consumption of 36 mg per day.^3^ Height *z* scores were −0.21 (95% CI, −0.41 to −0.02) lower in the fourth quartile compared with the first (Table 2) across ages 4 to 8 years. At age 7, this difference in *z* score translated to an approximate 1.5 cm difference. Weight *z* scores were −0.27 (95% CI, −0.47 to −0.07) lower in the third quartile compared with the first (Table 2), translating to an approximate 1.1 kg difference at age 7 years. There were no observed differences in BMI *z* scores, child fat mass index, or fat percentage. Findings were similar for paraxanthine quartiles, such that the children of women in the fourth quartile had smaller height *z* scores than those in the first quartile (β = −0.20; 95% CI, −0.38 to −0.01) (Table 2). There was no evidence of increased risk of overweight or obesity in any quartile of caffeine or paraxanthine.

**Table 2.** Mean Differences in Child Growth Parameters at Ages 4 to 8 Years, Comparing Quartiles of Maternal Plasma Caffeine and Paraxanthine, Environmental Influences of Child Outcomes Cohort of the National Institute of Child Health and Human Development Fetal Growth Studies^a^

Growth parameter (N = 788 children)	β (95% CI)							
	Caffeine quartiles, ng/mL				Paraxanthine quartiles, ng/mL			
	<25.4	25.4-146.6	146.7-575.2	≥575.3	<13.4	13.4-68.2	68.3-213.7	≥213.8
Body mass index *z* score	1 [Reference]	−0.13 (−0.33 to 0.08)	−0.25 (−0.45 to 0.04)	0.01 (−0.20 to 0.21)	1 [Reference]	−0.05 (−0.25 to 0.16)	−0.04 (−0.25 to 0.17)	−0.08 (−0.29 to 0.13)
Weight *z* score	1 [Reference]	−0.20 (−0.39 to 0.00)	−0.27 (−0.47 to −0.07)	−0.12 (−0.32 to 0.08)	1 [Reference]	−0.08 (−0.27 to 0.11)	−0.09 (−0.28 to 0.11)	−0.16 (−0.35 to 0.04)
Height *z* score	1 [Reference]	−0.18 (−0.37 to −0.01)	−0.19 (−0.38 to −0.01)	−0.21 (−0.41 to −0.02)	1 [Reference]	−0.09 (−0.27 to 0.09)	−0.14 (−0.32 to 0.05)	−0.20 (−0.38 to −0.01)
Fat mass index, kg/m^2^	1 [Reference]	0.02 (−0.42 to 0.47)	−0.12 (−0.57 to 0.32)	0.04 (−0.41 to 0.49)	1 [Reference]	0.07 (−0.37 to 0.51)	0.10 (−0.34 to 0.55)	−0.18 (−0.62 to 0.27)
Fat mass percentile	1 [Reference]	0 (−0.02 to 0.02)	−0.01 (−0.03 to 0.01)	0 (−0.02 to 0.02)	1 [Reference]	0 (−0.02 to 0.02)	0 (−0.02 to 0.02)	−0.01 (−0.03 to 0.01)
Risk of overweight or obesity^b^	1 [Reference]	0.88 (0.62 to 1.24)	0.71 (0.49 to 1.01)	0.94 (0.66 to 1.34)	1 [Reference]	0.87 (0.62 to 1.21)	0.87 (0.61 to 1.24)	0.87 (0.61 to 1.25)

^a^ Analyses were adjusted for maternal age, race, education, marital status, parity, body mass index, and cotinine.

^b^ Overweight and obesity were calculated using cut points established by the International Obesity Task Force.^17^

### CPP Cohort

**Figure 1.**
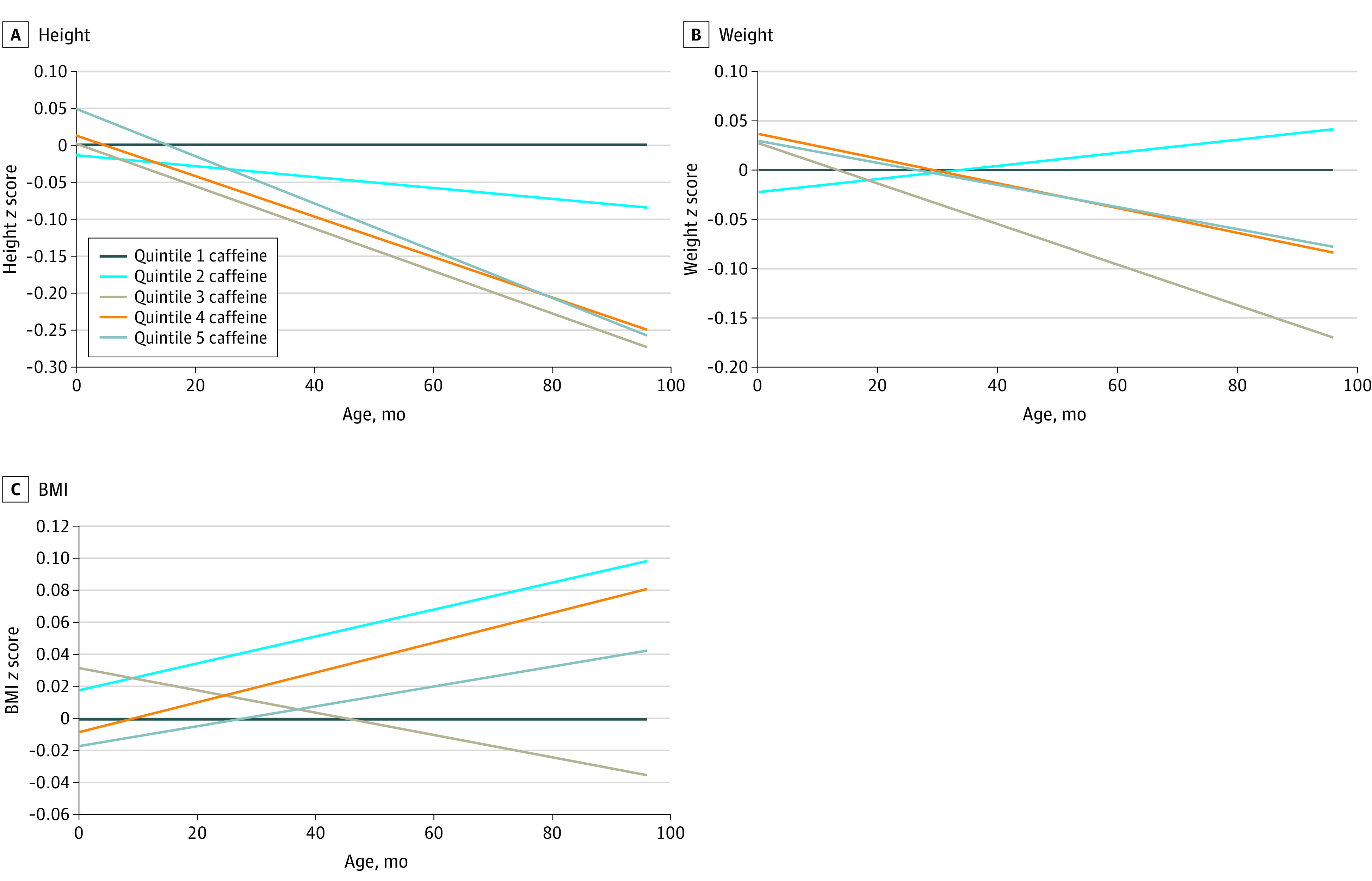
Differences in Estimated Mean *z* Scores for Height, Weight, and Body Mass Index (BMI) From Birth to 8 Years by Caffeine Quintile, in the Collaborative Perinatal Project Linear mixed models were used to create trajectories of height, weight, and BMI and estimated mean *z* scores at birth; 4, 8, and 12 months; and 3, 4, 7, and 8 years (corresponding to the targeted study visits). The differences were calculated by subtracting quintiles 2 through 5 from quintile 1 (referent group) and a scatterplot created with Loess lines presented. Serum caffeine quintiles are defined as follows: 1, less than 63.7 ng/mL; 2, 63.7 to 407.3 ng/mL; 3, 407.4 to 1197.1 ng/mL; 4, 1197.2 to 2594.4 ng/mL; and 5, greater than or equal to 2594.5 ng/mL.

**Figure 2.**
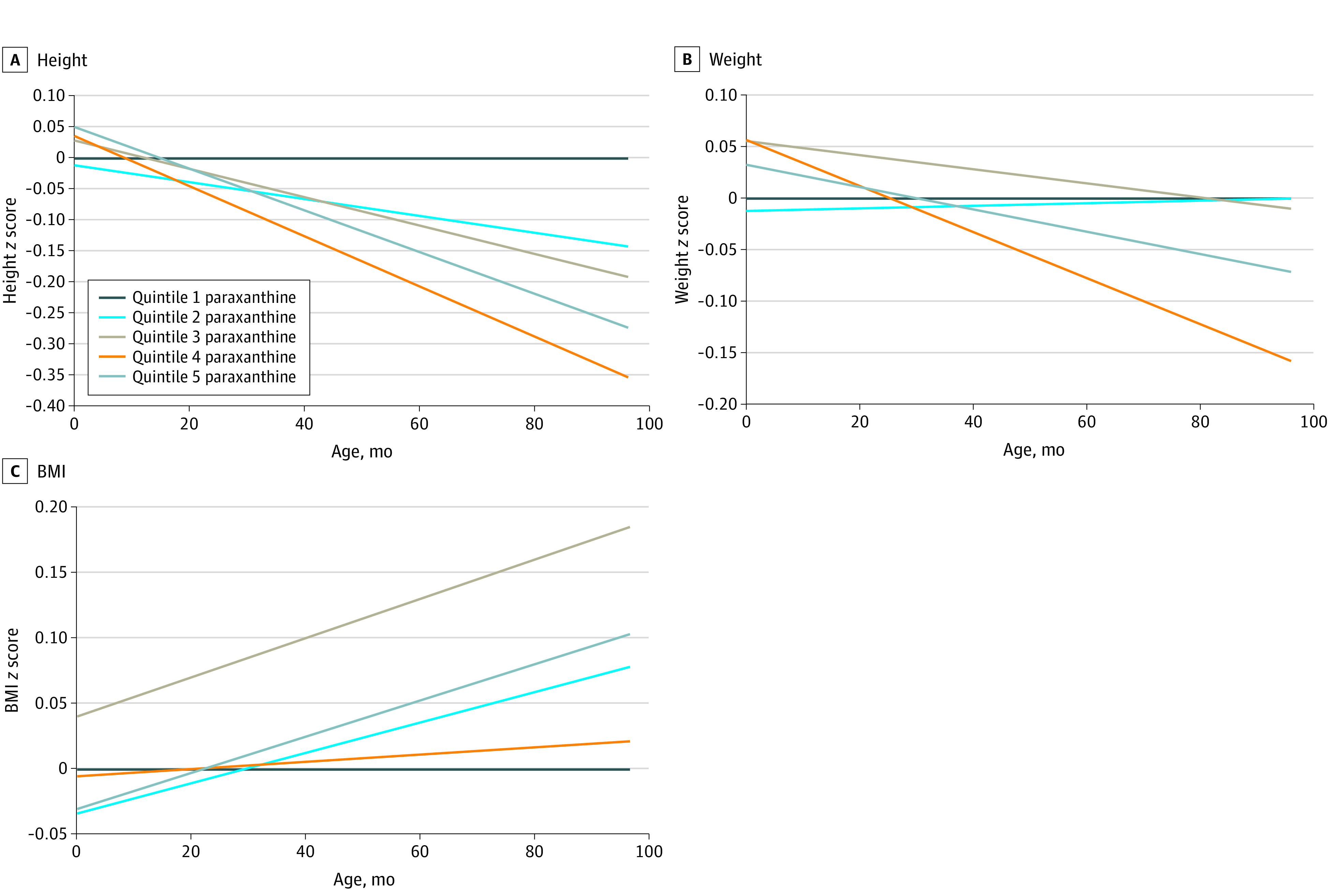
Differences in Estimated Mean *z* Scores for Height, Weight, and Body Mass Index (BMI) From Birth to 8 Years by Paraxanthine Quintile, in the Collaborative Perinatal Project Linear mixed models were used to create trajectories of height, weight, and BMI and estimated mean *z* scores at birth; 4, 8, and 12 months; and 3, 4, 7, and 8 years (corresponding to the targeted study visits). The differences were calculated by subtracting quintiles 2 through 5 from quintile 1 (referent group) and a scatterplot created with Loess lines presented. Serum paraxanthine quintiles are defined as follows: 1, less than 63.7 ng/mL; 2, 63.7 to 407.3 ng/mL; 3, 407.4 to 1197.1 ng/mL; 4, 1197.2 to 2594.4 ng/mL; and 5, greater than or equal to 2594.5 ng/mL.

The median (IQR) caffeine and paraxanthine concentrations were 625.5 (86.2 to 1994.7) ng/mL and 295.9 (70.2 to 666.8) ng/mL, respectively, which roughly translates to median consumption of 2 cups per day of coffee.^22^ Compared with the lowest quintile (Figure 1 and eTable 2 in the Supplement), children of women in the highest quintile of caffeine had consistently lower height *z* scores, with a widening gap through age 8 years (β at 4 years = −0.16 [95% CI, −0.31 to −0.01]; β at 5 years = −0.21 [95% CI, −0.37 to −0.05]; β at 7 years = −0.32 [95% CI, −0.50 to −0.13]; β at 8 years = −0.37 [95% CI, −0.57 to −0.16]), translating to a 0.68 to 2.2 cm difference in height from ages 4 to 8 years. Beginning at age 5 years, there were lower weight *z* scores in the third quintile (age 5 years β = −0.16 [95% CI, −0.41 to −0.03]; age 8 years β = −0.22 [95% CI, −0.41 to −0.03]) (Figure 1). There was no difference in BMI *z* scores across quintiles of caffeine concentrations, with estimates ranging from −0.03 to 0.15 (Figure 1). Results were similar for paraxanthine, with significant differences in height *z* scores beginning at 3 years in the fifth quintile (β = −0.16; 95% CI, −0.30 to −0.01) and becoming most pronounced by age 8 years (β = −0.39; 95% CI, −0.60 to −0.18) (Figure 2 and eTable 3 in the Supplement). Child weight *z* scores did not significantly differ in any quintile at any age. BMI *z* score estimates tended to be higher in higher quintiles, but were only significant for the second and third quintiles, ranging from 0.22 to 0.26 at ages 7 and 8 years. The risk of overweight or obesity was higher for the fourth and fifth quintiles of caffeine and paraxanthine but did not reach the level of significance (eTables 2 and 3 in the Supplement). There were no significant interactions with child sex, indicating that associations did not vary by child sex.

## Discussion

In this cohort study, higher maternal caffeine and paraxanthine concentrations were associated with shorter stature persisting up to age 8 years in 2 pregnancy cohorts with longitudinal follow-up and distinct patterns of low and high caffeine consumption. Serum and plasma caffeine were inversely associated with child weight in the third quartile and quintile only, whereas no clear patterns of association were observed between maternal caffeine and child BMI. Though maternal caffeine and paraxanthine concentrations were generally not associated with risk of overweight or obesity, our findings indicate that maternal caffeine consumption is associated with long-term decreases in child height. This association occurred even with maternal consumption below current recommendations of 200 mg day.^23^

Though we did not find strong associations between increasing maternal caffeine consumption and higher childhood BMI, our findings are consistent with prior studies.^6,7,8,9,24^ We consistently observed shorter height, which has been associated with increased risk of multiple cardiometabolic diseases in both pregnant and nonpregnant individuals.^25,26^ Shorter stature, though not necessarily associated with obesity in childhood, has been associated with obesity and increased diabetes risk in adults.^27^ Shorter height may also contribute to higher BMI, similar to a study^7^ that found small differences in BMI *z* scores in conjunction with shorter height, but not necessarily higher weight. In the study by Voerman et al,^7^ compared with the low maternal caffeine consumption group (<2 cups of coffee per day), weight *z* scores were lower over time in the group with consumption of 2 to 3.9 cups (approximately 180-359 mg), whereas weight *z* scores were higher over time in the highest consumption groups (≥400 mg). We observed a similar phenomenon in the low caffeine consumption ECHO-FGS cohort, where weight *z* scores were lower in the third quartile of caffeine concentrations. Though we and other researchers have observed no threshold effect of caffeine on birth weight,^1,3^ our findings and the Voerman study^7^ suggest there may be a threshold effect of maternal consumption for offspring child weight—that is, lower consumption may not be associated with child weight gain and may be associated with lower weight, as observed in the CPP, where weight was lower in the third quintile but not higher quintiles of caffeine. Nearly all prior studies of caffeine and child growth had higher mean consumption than the mothers of children in the ECHO-FGS cohort, which may explain our null finding for fat mass measures in ECHO-FGS. Prior studies^7,8^ have only found increased fat mass and adiposity among offspring whose mothers consumed 4 or more cups of coffee per day compared with those who consumed less than 2 cups per day.

Though decreased child height to weight could contribute to increased risk of overweight or obesity, we did not find strong evidence for an association between caffeine and risk of child overweight or obesity, which is consistent with another study^27^ that found no increased risk of obesity in children with shorter height. Lack of an observed association in ECHO-FGS could be owing to lower overall caffeine consumption. A recent study found that the risk of overweight or obesity was lower for children whose mothers consumed 200 to 300 mg of caffeine per day, but increased with each higher-order category of consumption.^8^ This finding is consistent with our observation of lower but imprecise estimates of risk for overweight or obesity for children in caffeine categories that likely translate to less than 200 mg per day of consumption.^22^ In the CPP cohort, estimates of risk for child overweight in the fourth and fifth quintiles of caffeine and paraxanthine concentrations were similar to those observed in the highest category of caffeine consumption (≥6 cups of coffee) in a Dutch study.^7^ Though results in both the CPP and the Dutch study were not statistically significant, they are consistent in terms of magnitude of the estimates trending toward elevated risk of overweight or obesity.

An association between maternal caffeine consumption and abnormal child growth, mainly decreased child height, is biologically plausible given that caffeine and paraxanthine cross the placenta.^28^ Maternal caffeine metabolism tends to slow progressively over pregnancy, and the fetus and placenta do not produce CYP450, the primary enzyme required for caffeine metabolism.^28^ Maternal caffeine consumption may also increase maternal glucocorticoid circulation while inhibiting fetal regulatory processes that break down glucocorticoids.^4,29^ These factors contribute to an accumulation of caffeine and its metabolites in fetal tissues that could disrupt normal fetal growth via alterations in the hypothalamic-pituitary-adrenal axis or increased fetal insulin sensitivity from increased glucocorticoid exposure.^4^ This mechanism, along with associations between maternal caffeine measures and subsequent fetal and child growth, are consistent with the developmental origins of health and disease paradigm.^30^ Suboptimal fetal growth has been associated with metabolic changes that may increase risk of excess weight gain in infancy, childhood obesity, and chronic cardiometabolic disease, though specific effects on child height have not been explored.^6,7,8,29^ In our prior study^31^ of caffeine and neonatal anthropometry, we observed decreased lean mass measures, but not fat mass, which could increase the risk of future metabolic disturbance.

Though the clinical implications of an approximately 2-cm height difference are unclear, our findings for height are similar in magnitude to those of children whose mothers smoked during pregnancy. Longitudinal cohort studies^32,33^ have demonstrated differences in height *z* scores ranging from −0.19 to −0.51, compared with the offspring of nonsmokers. Future research in caffeine consumption during pregnancy should follow child growth into puberty and beyond to determine whether height gaps continue to widen into adulthood, and whether shorter height associated with maternal caffeine consumption confers greater risk for cardiometabolic dysfunction.

### Limitations and Strengths

This study has limitations that should be noted. We did not have sufficient information on some potential confounding factors, such as maternal diet, nausea or vomiting during pregnancy, or paternal height, though it is unlikely any of these factors would fully explain our findings. Because the CPP is a historical cohort of children with growth assessed in the 1960s and 1970s, we cannot account for other confounding factors unique to the time period and social norms that may have influenced maternal lifestyle factors. However, in a negative control study^6^ of more than 50 000 women, researchers found a stronger association between maternal caffeine intake and risk of child obesity at age 3 years compared with partner intake of caffeine at the time of pregnancy, with similar findings in a smaller negative control study, supporting the strong role of intrauterine factors in this association vs lifestyle factors.^24^ Altogether, the consistency in results between the cohorts across analyses supports an association between caffeine and child height that begins in utero. We had child growth measures at only 1 time point for ECHO-FGS, though given the consistency with height, weight, and BMI *z* scores observed at all time points in the CPP, we do not believe the overall interpretation of our findings would change appreciably with multiple measures from ECHO-FGS. It is also unclear how well measured caffeine and paraxanthine reflect habitual caffeine consumption. Previous studies^34^ have identified that consumption habits are consistent in reproductive-aged women and remain stable across pregnancy. We have previously observed similar results for both self-reported and measured caffeine and paraxanthine.^3^ Thus, we have good confidence that measured first trimester caffeine and paraxanthine is an adequate proxy for habitual intake on a population level, without the limitations of self-reported intake.^35^

A strength of our study is the use of 2 independent cohorts to evaluate associations: a contemporary cohort reflecting modern caffeine consumption trends in the US and a historical cohort with high consumption. Our findings among the ECHO-FGS cohort are novel and important because they demonstrate that the inverse associations between maternal caffeine consumption and child height persist into childhood even in a low-consumption sample when prior associations have only been observed in cohorts with higher maternal consumption. Furthermore, by including a historical cohort, we demonstrated that the results are robust across time periods and across different levels of caffeine consumption. Though it is challenging to translate biomarker concentrations of caffeine back to milligrams per day consumed, according to prior estimates, caffeine concentrations for the fourth quartile equated to less than 150 mg per day,^22^ and women from ECHO-FGS reported consuming an average of 36 mg per day.^3^ We also had objective measures of caffeine and paraxanthine, as opposed to self-reported measures, that may not include all sources of caffeine, and may improperly estimate variations in caffeine based on preparation or serving size. Our child growth parameters were rigorously collected, following standard protocols at study visits and checked for improbable values, with multiple points of longitudinal measures from birth to age 8 years for the CPP cohort. We also included a more racially and ethnically diverse sample than prior studies.

## Conclusions

In this retrospective cohort analysis of caffeine consumption during pregnancy and longitudinal child growth, maternal caffeine consumption even in amounts lower than currently recommended guidelines of less than 200 mg per day during pregnancy was associated with smaller child height beginning at age 4 years and persisting to age 8 years. The clinical implication of this height difference is unclear and warrants future investigation.

## Notes

Supplement. **eMethods.** Collaborative Perinatal Project (CPP) Overview and Additional Analytic Considerations **eTable 1.** Maternal and Child Characteristics by Quintile (Qn) of Serum Caffeine Concentration, Collaborative Perinatal Project, n=1,622 **eTable 2.** Mean Differences in Height, Weight, and BMI Z Scores, Comparing Quintiles (Qn) of Maternal Serum Caffeine, Collaborative Perinatal Project (n=1,622) **eTable 3.** Mean Differences in Height, Weight, and BMI Z Scores, Comparing Quintiles (Qn) of Maternal Serum Paraxanthine, Collaborative Perinatal Project (n=1,622)
eReferences
