# Error in Table 1

**DOI:** 10.1001/jamanetworkopen.2022.47489

**Published:** 2022-12-01

In the Original Investigation titled “Association of Maternal Caffeine Consumption During Pregnancy With Child Growth,”^1^ published October 31, 2022, there was an error in Table 1. In the second column, maternal height should have been 1.63 (0.07) m. This article has been corrected.^1^
