# Supplementary material for: Association of Maternal Caffeine Consumption During Pregnancy With Child Growth
Source: JAMA Netw Open. 2022 Oct 31;5(10):e2239609. doi: 10.1001/jamanetworkopen.2022.39609 (PMC9623443; doi:10.1001/jamanetworkopen.2022.39609)
Supplement: Supplement. — eMethods. Collaborative Perinatal Project (CPP) Overview and Additional Analytic Considerations eTable 1. Maternal and Child Characteristics by Quintile (Qn) of Serum Caffeine Concentration, Collaborative Perinatal Project, n=1,622 eTable 2. Mean Differences in Height, Weight, and BMI Z Scores, Comparing Quintiles (Qn) of Maternal Serum Caffeine, Collaborative Perinatal Project (n=1,622) eTable 3. Mean Differences in Height, Weight, and BMI Z Scores, Comparing Quintiles (Qn) of Maternal Serum Paraxanthine, Collaborative Perinatal Project (n=1,622) eReferences [file jamanetwopen-e2239609-s001.pdf]

Supplemental Online Content

Gleason JL, Sundaram R, Mitro SD, et al. Association of maternal caffeine consumption during pregnancy with child growth. *JAMA Netw Open*. 2022;5(10):e2239609. doi:10.1001/jamanetworkopen.2022.39609

eMethods. Collaborative Perinatal Project (CPP) Overview and Additional Analytic Considerations

eTable 1. Maternal and Child Characteristics by Quintile (Qn) of Serum Caffeine Concentration, Collaborative Perinatal Project, n=1,622

eTable 2. Mean Differences in Height, Weight, and BMI Z Scores, Comparing Quintiles (Qn) of Maternal Serum Caffeine, Collaborative Perinatal Project (n=1,622)

eTable 3. Mean Differences in Height, Weight, and BMI Z Scores, Comparing Quintiles (Qn) of Maternal Serum Paraxanthine, Collaborative Perinatal Project (n=1,622)

eReferences

This supplemental material has been provided by the authors to give readers additional information about their work.

© 2022 Gleason JL et al. *JAMA Network Open.*

eMethods. Collaborative Perinatal Project (CPP) Overview and Additional Analytic Considerations

The Collaborative Perinatal Project (CPP) was a prospective cohort of pregnant women recruited from 12 US clinical sites (1959-1965). Their offspring were followed up with physical and developmental assessments through 8 years.[1] The present analysis included CPP participants selected as controls for a previous case-control study of serum caffeine and paraxanthine and spontaneous abortion.[2] Children were eligible for this analysis if they were singletons with height or weight data for at least one follow-up visit (n=2,727). Due to the strong confounding effect of smoking,[3] and to be consistent with ECHO-FGS, which includes primarily nonsmokers, we further limited our sample to children of nonsmokers (n=1,622). Though the CPP represents a different historical period, it is advantageous for the present study because of its repeated assessments of early childhood growth. Trained research staff weighed and measured infants and children with calibrated scales and stadiometers, recording recumbent length up to 20 months and standing height thereafter. Children's height and weight was assessed at birth and at follow-up visits at 4, 8, and 12 months, and 3, 4, 7, and 8 years.[1] In the CPP, the mean (range) number of anthropometric measures per child was 5 (1-11), and 41.7% (n=1,126) exceeded the threshold for excess infant weight gain, while 10.8% were overweight or obese at any age between 2-8.

To evaluate associations between caffeine and paraxanthine quintiles and child growth we fit generalized linear mixed models for each growth parameter—height, weight, and BMI z-scores. To calculate risk of overweight/obesity, we fit Poisson regression models with a robust error variance. For the CPP, these models included random intercepts for each child to account for correlation of repeated measures over time and nested maternal intercepts to account for repeat pregnancies.

© 2022 Gleason JL et al. *JAMA Network Open.*

eTable 1. Maternal and Child Characteristics by Quintile (Qn) of Serum Caffeine Concentration, Collaborative Perinatal Project, n=1,622

	Overall (n=1622) No. (%)	Qn1 <63.7 ng/mL (n=331) No. (%)	Qn2 63.7-407.3 ng/mL (n=318) No. (%)	Qn3 407.4-1197.1 ng/mL (n=325) No. (%)	Qn4 1197.2-2594.4 ng/mL (n=324) No. (%)	Qn5 ≥2594.5 ng/mL (n=324) No. (%)
**Maternal characteristics**[a]						
Age, years, mean (SD)	25.3 (5.9)	23.5 (5.5)	24.4 (5.8)	25.3 (5.6)	26.0 (5.7)	27.4 (6.1)
Socioeconomic index[b]						
0-3.9	379 (23.7)	105 (32.2)	63 (20.1)	74 (22.8)	67 (21.1)	70 (21.9)
4-5.9	401 (25.1)	76 (23.3)	82 (26.2)	68 (21.0)	82 (25.8)	93 (29.2)
6-7.9	415 (25.9)	79 (24.2)	82 (26.2)	92 (28.4)	85 (26.7)	77 (24.1)
8-9.9	405 (25.3)	66 (20.3)	86 (27.5)	90 (27.8)	84 (26.4)	79 (24.8)
Parity (pre-delivery)						
0	586 (36.2)	155 (47.0)	125 (39.4)	118 (36.4)	114 (35.2)	74 (22.9)
1	397 (24.5)	75 (22.7)	81 (25.6)	84 (25.9)	72 (22.2)	85 (26.3)
2	264 (16.3)	42 (12.7)	44 (13.9)	58 (17.9)	58 (17.9)	62 (19.2)
3+	371 (22.9)	58 (17.6)	67 (21.1)	64 (19.8)	80 (24.7)	102 (31.6)
Race/Ethnicity						
Black	525 (32.4)	171 (51.7)	111 (34.9)	106 (36.6)	76 (23.5)	61 (18.8)
White	972 (59.9)	149 (45.0)	189 (59.4)	196 (60.3)	218 (67.3)	220 (67.9)
Other[c]	125 (7.7)	11 (8.8)	18 (5.7)	23 (7.1)	30 (9.3)	43 (13.3)
Married	1468 (90.5)	277 (83.7)	280 (88.1)	302 (92.9)	301 (92.9)	308 (95.1)
Maternal pre-pregnancy BMI, mean (SD)	22.7 (4.0)	22.6 (4.0)	22.5 (3.3)	22.9 (4.1)	22.8 (4.5)	22.9 (4.1)
Child sex, male, n (%)	805 (49.7)	158 (47.9)	152 (47.8)	169 (52.2)	160 (49.5)	166 (51.2)

[a] Includes liveborn singletons with at least one growth measurement and caffeine or paraxanthine measure; Missing data: socioeconomic index=22 (1.3%), Parity=4 (0.2%), Maternal BMI=74 (2.7%)

[b] Socioeconomic index combines parental education, employment, and household income and is interpreted as a percentile score of socioeconomic status.[4]

[c] Other race includes Asian, Puerto Rican, and self-reported "Other." Categories combined due to small numbers in each group.

© 2022 Gleason JL et al. *JAMA Network Open.*

eTable 2. Mean Differences in Height, Weight, and BMI Z Scores, Comparing Quintiles (Qn) of Maternal Serum Caffeine, Collaborative Perinatal Project (n=1,622)[a]

Growth Parameter	Age	Qn1 <63.7 ng/mL	Qn2 63.7-407.3 ng/mL	Qn3 407.4-1197.1 ng/mL	Qn4 1197.2-2594.4 ng/mL	Qn5 ≥2594.5 ng/mL
Weight Z [b]	0	[Reference]	-0.01 (-0.16 to 0.13)	-0.06 (-0.21 to 0.08)	-0.03 (-0.18 to 0.11)	-0.01 (-0.16 to 0.14)
	4	[Reference]	-0.01 (-0.15 to 0.13)	-0.07 (-0.21 to 0.07)	-0.04 (-0.18 to 0.10)	-0.02 (-0.16 to 0.13)
	8	[Reference]	-0.01 (-0.15 to 0.12)	-0.07 (-0.21 to 0.06)	-0.04 (-0.18 to 0.10)	-0.03 (-0.17 to 0.12)
	12	[Reference]	-0.01 (-0.15 to 0.12)	-0.08 (-0.22 to 0.05)	-0.04 (-0.18 to 0.09)	-0.03 (-0.17 to 0.11)
	36	[Reference]	-0.01 (-0.14 to 0.12)	-0.12 (-0.25 to 0.01)	-0.07 (-0.20 to 0.06)	-0.07 (-0.21 to 0.07)
	48	[Reference]	-0.01 (-0.14 to 0.13)	-0.14 (-0.28 to 0.00)	-0.08 (-0.22 to 0.06)	-0.09 (-0.24 to 0.05)
	60	[Reference]	-0.01 (-0.15 to 0.14)	**-0.16 (-0.31 to -0.01)**	-0.10 (-0.25 to 0.05)	-0.11 (-0.26 to 0.04)
	84	[Reference]	0.00 (-0.18 to 0.17)	**-0.20 (-0.37 to -0.02)**	-0.12 (-0.31 to 0.06)	-0.15 (-0.33 to 0.03)
	96	[Reference]	0.00 (-0.19 to 0.19)	**-0.22 (-0.41 to -0.03)**	-0.13 (-0.34 to 0.07)	-0.17 (-0.37 to 0.03)
Height Z [c]	0	[Reference]	0.03 (-0.14 to 0.19)	-0.06 (-0.22 to 0.10)	-0.07 (-0.23 to 0.09)	0.04 (-0.13 to 0.21)
	4	[Reference]	0.02 (-0.14 to 0.18)	-0.07 (-0.23 to 0.09)	-0.08 (-0.24 to 0.07)	0.02 (-0.14 to 0.19)
	8	[Reference]	0.01 (-0.15 to 0.16)	-0.08 (-0.24 to 0.07)	-0.10 (-0.25 to 0.06)	0.01 (-0.16 to 0.17)
	12	[Reference]	0.00 (-0.15 to 0.15)	-0.09 (-0.24 to 0.06)	-0.11 (-0.26 to 0.04)	-0.01 (-0.17 to 0.15)
	36	[Reference]	-0.06 (-0.20 to 0.08)	-0.16 (-0.30 to -0.02)	-0.18 (-0.32 to -0.04)	-0.11 (-0.26 to 0.04)
	48	[Reference]	-0.08 (-0.22 to 0.05)	**-0.20 (-0.34 to -0.05)**	**-0.21 (-0.36 to -0.07)**	**-0.16 (-0.31 to -0.01)**
	60	[Reference]	-0.11 (-0.26 to 0.03)	**-0.23 (-0.38 to -0.08)**	**-0.25 (-0.40 to -0.10)**	**-0.21 (-0.37 to -0.05)**
	84	[Reference]	-0.17 (-0.34 to 0.01)	**-0.30 (-0.48 to -0.12)**	**-0.32 (-0.50 to -0.14)**	**-0.32 (-0.50 to -0.13)**
	96	[Reference]	-0.19 (-0.39 to 0.00)	**-0.33 (-0.53 to -0.14)**	**-0.36 (-0.56 to -0.16)**	**-0.37 (-0.57 to -0.16)**
BMI Z [d]	0	[Reference]	-0.03 (-0.17 to 0.12)	-0.01 (-0.15 to 0.13)	-0.05 (-0.19 to 0.09)	-0.02 (-0.17 to 0.12)
	4	[Reference]	-0.02 (-0.16 to 0.12)	-0.01 (-0.15 to 0.13)	-0.04 (-0.18 to 0.10)	-0.02 (-0.16 to 0.12)
	8	[Reference]	-0.01 (-0.15 to 0.12)	-0.01 (-0.14 to 0.13)	-0.03 (-0.17 to 0.10)	-0.01 (-0.15 to 0.13)
	12	[Reference]	-0.01 (-0.14 to 0.12)	-0.01 (-0.14 to 0.12)	-0.02 (-0.15 to 0.11)	-0.01 (-0.15 to 0.13)
	36	[Reference]	0.03 (-0.10 to 0.16)	0.00 (-0.12 to 0.12)	0.03 (-0.10 to 0.15)	0.02 (-0.12 to 0.15)
	48	[Reference]	0.05 (-0.08 to 0.18)	0.00 (-0.13 to 0.13)	0.05 (-0.09 to 0.19)	0.03 (-0.11 to 0.17)
	60	[Reference]	0.07 (-0.07 to 0.21)	0.00 (-0.13 to 0.14)	0.08 (-0.07 to 0.23)	0.04 (-0.11 to 0.19)
	84	[Reference]	0.11 (-0.07 to 0.28)	0.01 (-0.16 to 0.18)	0.12 (-0.06 to 0.31)	0.07 (-0.12 to 0.25)
	96	[Reference]	0.13 (-0.07 to 0.32)	0.01 (-0.18 to 0.20)	0.15 (-0.06 to 0.36)	0.08 (-0.12 to 0.29)
RR (95% CI)	Overweight/ Obesity	[Reference]	1.11 (0.73 to 1.70)	0.83 (0.54 to 1.29)	1.23 (0.83 to 1.81)	1.13 (0.75 to 1.70)

[a] Predicted mean differences calculated using linear mixed models with interactions by child age and caffeine, adjusted for maternal age, race, socioeconomic index, marital status, parity, smoking status, and study site. Number of measures recorded at study visits include: birth=1,622, 4 months=1,469, 8 months=768, 12 months=1,355, 3 years=534, 4 years=1,102, 7 years=1,284, 8 years=476; bolded cells represent estimates where $P<0.05$.

© 2022 Gleason JL et al. *JAMA Network Open.*

[b] Additionally adjusted for maternal prepregnancy weight
[c] Additionally adjusted for maternal height
[d] Additionally adjusted for maternal prepregnancy BMI

© 2022 Gleason JL et al. *JAMA Network Open.*

eTable 3. Mean Differences in Height, Weight, and BMI Z Scores, Comparing Quintiles (Qn) of Maternal Serum Paraxanthine, Collaborative Perinatal Project (n=1,622)[a]

Growth Parameter	Age	Qn1 <58.4 ng/mL	Qn2 58.4-243 ng/mL	Qn3 243.1-534.3 ng/mL	Qn4 534.4-1005.1 ng/mL	Qn5 ≥1005.2 ng/mL
Weight Z [b]	0	[Reference]	-0.02 (-0.16 to 0.13)	0.04 (-0.10 to 0.19)	-0.03 (-0.18 to 0.11)	-0.03 (-0.17 to 0.12)
	4	[Reference]	-0.01 (-0.15 to 0.13)	0.04 (-0.10 to 0.18)	-0.04 (-0.18 to 0.11)	-0.03 (-0.17 to 0.11)
	8	[Reference]	-0.01 (-0.15 to 0.13)	0.03 (-0.11 to 0.17)	-0.04 (-0.18 to 0.10)	-0.04 (-0.18 to 0.10)
	12	[Reference]	0.00 (-0.14 to 0.13)	0.03 (-0.11 to 0.16)	-0.04 (-0.18 to 0.09)	-0.04 (-0.18 to 0.09)
	36	[Reference]	0.03 (-0.11 to 0.16)	0.00 (-0.13 to 0.12)	-0.06 (-0.19 to 0.07)	-0.08 (-0.22 to 0.05)
	48	[Reference]	0.04 (-0.10 to 0.18)	-0.02 (-0.15 to 0.11)	-0.07 (-0.21 to 0.06)	-0.10 (-0.24 to 0.04)
	60	[Reference]	0.05 (-0.10 to 0.20)	-0.04 (-0.18 to 0.10)	-0.08 (-0.22 to 0.06)	-0.12 (-0.27 to 0.03)
	84	[Reference]	0.08 (-0.10 to 0.27)	-0.07 (-0.23 to 0.10)	-0.10 (-0.27 to 0.07)	-0.16 (-0.34 to 0.02)
	96	[Reference]	0.10 (-0.11 to 0.30)	-0.08 (-0.27 to 0.10)	-0.11 (-0.30 to 0.08)	-0.18 (-0.37 to 0.02)
Height Z [c]	0	[Reference]	0.00 (-0.17 to 0.17)	0.04 (-0.12 to 0.20)	-0.01 (-0.17 to 0.16)	-0.02 (-0.18 to 0.15)
	4	[Reference]	-0.01 (-0.17 to 0.16)	0.03 (-0.13 to 0.18)	-0.02 (-0.18 to 0.14)	-0.03 (-0.19 to 0.13)
	8	[Reference]	-0.01 (-0.17 to 0.14)	0.01 (-0.14 to 0.16)	-0.04 (-0.20 to 0.12)	-0.05 (-0.20 to 0.11)
	12	[Reference]	-0.02 (-0.18 to 0.13)	0.00 (-0.15 to 0.15)	-0.05 (-0.21 to 0.10)	-0.06 (-0.21 to 0.09)
	36	[Reference]	-0.07 (-0.21 to 0.07)	-0.08 (-0.22 to 0.06)	**-0.14 (-0.28 to 0.00)**	**-0.16 (-0.30 to -0.01)**
	48	[Reference]	-0.09 (-0.23 to 0.05)	-0.12 (-0.27 to 0.02)	**-0.19 (-0.33 to -0.05)**	**-0.20 (-0.35 to -0.05)**
	60	[Reference]	-0.11 (-0.26 to 0.04)	**-0.17 (-0.32 to -0.02)**	**-0.24 (-0.38 to -0.09)**	**-0.25 (-0.41 to -0.09)**
	84	[Reference]	-0.16 (-0.34 to 0.02)	**-0.25 (-0.42 to -0.07)**	**-0.33 (-0.50 to -0.16)**	**-0.34 (-0.53 to -0.15)**
	96	[Reference]	-0.18 (-0.38 to 0.01)	**-0.29 (-0.48 to -0.10)**	**-0.37 (-0.56 to -0.19)**	**-0.39 (-0.60 to -0.18)**
BMI Z [d]	0	[Reference]	-0.04 (-0.18 to 0.11)	0.00 (-0.14 to 0.14)	-0.07 (-0.21 to 0.08)	-0.02 (-0.16 to 0.13)
	4	[Reference]	-0.02 (-0.16 to 0.11)	0.01 (-0.13 to 0.15)	-0.06 (-0.20 to 0.08)	-0.01 (-0.15 to 0.13)
	8	[Reference]	-0.01 (-0.15 to 0.12)	0.02 (-0.12 to 0.15)	-0.05 (-0.19 to 0.09)	-0.01 (-0.14 to 0.13)
	12	[Reference]	0.00 (-0.13 to 0.13)	0.02 (-0.11 to 0.15)	-0.04 (-0.17 to 0.09)	0.00 (-0.14 to 0.13)
	36	[Reference]	0.07 (-0.05 to 0.20)	0.07 (-0.05 to 0.20)	0.02 (-0.11 to 0.14)	0.03 (-0.10 to 0.16)
	48	[Reference]	0.11 (-0.02 to 0.24)	0.10 (-0.03 to 0.22)	0.04 (-0.09 to 0.18)	0.05 (-0.09 to 0.18)
	60	[Reference]	0.15 (0.00 to 0.29)	0.12 (-0.01 to 0.26)	0.07 (-0.07 to 0.22)	0.06 (-0.09 to 0.21)
	84	[Reference]	**0.22 (0.04 to 0.40)**	**0.17 (0.01 to 0.34)**	0.13 (-0.05 to 0.31)	0.09 (-0.09 to 0.28)
	96	[Reference]	**0.26 (0.06 to 0.46)**	**0.20 (0.01 to 0.38)**	0.16 (-0.04 to 0.36)	0.11 (-0.10 to 0.31)
RR (95% CI)	Overweight/ Obesity	[Reference]	1.38 (0.91, 2.11)	1.10 (0.73, 1.66)	1.20 (0.80, 1.81)	1.37 (0.91, 2.05)

[a] Predicted mean differences calculated using linear mixed models with interactions by child age and paraxanthine, adjusted for maternal age, race, socioeconomic index, marital status, parity, smoking status, and study site. Number of measures recorded at study visits include: birth=1,622, 4 months=1,469, 8 months=768, 12 months=1,355, 3 years=534, 4 years=1,102, 7 years=1,284, 8 years=476; bolded cells represent estimates where $P<0.05$.

© 2022 Gleason JL et al. *JAMA Network Open.*

[b] Additionally adjusted for maternal prepregnancy weight
[c] Additionally adjusted for maternal height
[d] Additionally adjusted for maternal prepregnancy BMI

© 2022 Gleason JL et al. *JAMA Network Open*.

eReferences

1. Klebanoff MA. The Collaborative Perinatal Project: a 50-year retrospective. *Paediatr Perinat Epidemiol.* 2009;23(1):2-8.
2. Klebanoff MA, Levine RJ, DerSimonian R, Clemens JD, Wilkins DG. Maternal serum paraxanthine, a caffeine metabolite, and the risk of spontaneous abortion. *N Engl J Med.* 1999;341(22):1639-1644.
3. Oken E, Levitan EB, Gillman MW. Maternal smoking during pregnancy and child overweight: systematic review and meta-analysis. *Int J Obes (Lond).* 2008;32(2):201-210.
4. Myrianthopoulos NC, French KS. An application of the U.S. Bureau of the Census socioeconomic index to a large, diversified patient population. *Soc Sci Med.* 1968;2(3):283-299.

© 2022 Gleason JL et al. *JAMA Network Open.*
